# Informal care and health behaviors among elderly people with chronic diseases

**DOI:** 10.1186/s41043-017-0117-x

**Published:** 2017-12-06

**Authors:** Hong Wu, Naiji Lu

**Affiliations:** School of Medicine and Health Management, Tongji Medical College, Huazhong University of Science and Technology, 13 Hangkong road, Qiaokou District, Wuhan, Hubei Province China

**Keywords:** Informal care, Health behaviors, Chronic diseases, Elderly people, CHARLS, Propensity score matching method

## Abstract

**Background:**

The mechanism by which social relationships influence health can be interpreted as a social network regulating one’s health behaviors. Based on the hypothesis that relatives, friends, or neighbors are sources of social support and may monitor one’s health behaviors, researchers have gotten significant and consistent results that a social network can regulate health behaviors. However, few empirical studies have been conducted to examine the role of informal care in the regulation of health behaviors, especially for elderly individuals with chronic diseases that can be controlled by healthy behaviors. This paper researched the effects of informal care on health behaviors—smoking control, dietetic regulation, weight control, and maintenance of exercise—among elderly patients with chronic diseases in China who are facing the challenge of aging.

**Methods:**

We used the propensity score matching method to control the impacts of a very rich set of family and individual characteristics. The 2011–2012 national baseline data of the China Health and Retirement Longitudinal Study (CHARLS) was used.

**Results:**

Our findings showed that informal care could significantly help improve the health behaviors of elderly people. Informal care could improve the compliance of smoking control and dietetic regulation significantly. Elderly people with informal care smoked less and consumed more meals per day. For weight control, informal care helped decrease the possibility of weight gain of elderly people, but its impacts were not significant for BMI and weight loss. Last, for the elders, informal care could only help increase the probability of walking exercise; however, there was no significant result for moderate exercise.

**Conclusions:**

Findings from this study highlight the importance of informal care among elderly people. Our results appeal to policy makers who aim to control chronic diseases that they should take informal care into account and provide appropriate policies to meet the demand of informal care for elderly people.

**Electronic supplementary material:**

The online version of this article (10.1186/s41043-017-0117-x) contains supplementary material, which is available to authorized users.

## Background

It is widely accepted that social network participation is associated with better health outcomes. Social support and social control are two interpersonal mechanisms that have been identified as having implications for people’s health behaviors [6, 16]. Informal care, as the main source of social support and control for the elderly people, can provide assistance, promote health-enhancing behaviors, and monitor and influence the individual’s health behaviors. Prior studies mainly focused on the differences of health behaviors between married and unmarried individuals [3]. For informal care, although it is generally considered to be effective in improving the health status of elderly people, its role in changing health behaviors is still unknown. Thus, this paper aims to address this knowledge gap by examining the differences of health behaviors between cared and non-cared individuals. For health behaviors, we focus on smoking control, dietetic regulation, weight control, and maintenance of exercise.

### Social support/control and health behaviors

Social support theories can be used to explain the role of informal care in accomplishing patient compliance. These theories emphasize that support for health change can be elicited from family members or close friends. The adherence of doctor advices is generally higher when family members are involved in the change processes of health behaviors [28]. Family members can assist patients by engaging in the targeted behaviors, such as walking with the patients who are told to engage in exercise [4], and they can also help change the eating habits of patients [20]. Family members can serve to remind individuals to engage in health behaviors, and this has been found to help improve adherence [28]. Umberson [30] proves that marriage relationship as a support for social control is beneficial to health because people often monitor and control their spouse’s health behaviors.

Informal care is one of the leading important sources of care for elderly people. Based on Grossman’s health capital theory [14, 15], informal care, as a health production input, would help accumulate the health capital, which could lead to better health outcomes. The valuation of informal care in illness cost has been examined [2]. Many studies focus on the effects of informal care on the health outcomes and the use of health services [5, 12, 25], and most of these studies have proved that informal care can improve the health outcomes of elderly people [29]. Wu et al. [33] have found that informal care can decrease falls and other accidents (e.g., traffic accidents) among elderly people. Current studies have shown that caregivers have poor health behaviors, such as less nutrition, less exercise, poor sleep, and lower physical activities, which lead to adverse health outcomes [13]. Care provision which caregivers engage in helps explain the mechanisms of health decline in caregivers [13]. In turn, we deduce that informal care could help improve the health lifestyle and enhance the physical activities of care receivers. However, to date, how informal care affects the health behaviors of care receivers has not been studied.

### Health behaviors and chronic diseases

The number of patients with chronic diseases has exceeded 260 million in 2015. Chronic diseases account for 86.6% of deaths and nearly 70% of the total burden of diseases in China.^1^ The progression of chronic conditions may later lead to debilitating diseases that require costly care. The prevalence of chronic diseases and the limits of clinical approaches emphasize the need of changing to a healthy lifestyle. Most of the chronic diseases are preventable. Researchers and governments have recognized the importance of modifiable factors for the prevention of chronic diseases [8, 19]. Smoking, poor nutrition, and less physical activities contribute to the high rates of chronic diseases among people. Improvement of health behaviors among people with chronic diseases is vital to increase longevity and enhance quality of life.

A healthy lifestyle is critically important for patients with chronic diseases [9]. Findings indicate that health behaviors, such as smoking, drinking, regular exercise, and reasonable diet, are among the most important behavioral determinants of health status [17, 18]. Healthy behaviors help reduce the severity and risk of disease recurrence, improve the quality of life, and extend life expectancy [1, 27]. For example, smoking cessation can lower the risk of heart attack by half [24]. Smoking and drinking are usually associated with emotional distress [11], and depressed people may pay less attention to their self-care [22]. People with chronic diseases are encouraged to engage in regular physical activities, such as walking. However, the data shows that the exercise of elderly people with chronic diseases is not at the optimal level and is less than that of peers without chronic diseases [34].

### Aging population in China

China is facing the challenge of an aging population, and the aging rate is accelerating [26]. According to the census in 2015, the number of elderly people aged 60 and over is more than 17 million and nearly 3 million people are in unhealthy conditions or cannot take care of themselves in life.^2^ The prevalence of chronic diseases in the elderly people is 4.2 times that of the whole population.^3^

Although behavior regulation, such as smoking control, weight control, and maintenance of exercise, is essential to improving health, many elderly people still do not engage in healthy behaviors. Approximately 22% of adults aged 45~64 are smokers [21], and many people have low frequency exercise [10].

## Methods

### Data

The data used in this paper comes from the China Health and Retirement Longitudinal Study (CHARLS) which is a new survey conducted by the China Center for Economic Research at Peking University [35]. The baseline national wave of CHARLS was being fielded in 2011, and the respondents will be followed up every 2 years. So far, there are two national waves that have been published. The data sets have the advantage in that they contain detailed information about health status, health behaviors, and care information which is difficult to obtain. Wave 2 (collected in 2013) does not provide detailed information to help judge the non-cared individuals, so we only used the data in wave 1 (collected in 2011). This survey collects individuals aged 45 and over in China. In this paper, we call these respondents “elderly people.” Details of sampling frames and methodology, weighting strategies, and questionnaires can be found on its website (http://charls.pku.edu.cn/zh-CN/page/about/CHARLS).

The sample for the current study included elderly people who are diagnosed with chronic diseases. We selected the subsample based on two sample selection criteria: (1) as the questionnaires only collected doctor’s advice information for elderly people with two kinds of chronic diseases—diabetes and hypertension—we selected a subsample of respondents with diabetes or hypertension; (2) in order to control the influence of personal health awareness, we only selected elderly people with doctor’s advices: smoking control, dietetic regulation, weight control, or maintenance of exercise. Finally, 3198, 4466, 3352, and 3747 respondents were included in our research for smoking control, dietetic regulation, weight control, and maintenance of exercise, respectively.

### Measures

Informal care was measured by a dummy variable based on the question: “who most often helps you with dressing, bathing, eating, getting out of bed, using the toilet, controlling urination and defecation, doing chores, preparing hot meals, shopping, managing money, making phone calls, taking medications (Each respondent may choose up to 3 persons).” We took elderly people with informal care as the treated group and elderly people who did not get any help as the control group.

We examined four doctor’s advices: smoking control, dietetic regulation, weight control, and maintenance of exercise. The corresponding health behaviors were as follows: for smoking status, we included “the number of cigarettes consumed in one day” (*No.cigarette*) and “the time lag between wake up and the first cigarette” (*Wake smoking*); for dietetic regulation, we got “the number of meals the respondent normally ate per day” (*No.meals*); for weight control, we included BMI, “whether had gained (*Weight gain*) or lost (*Weight loss*) 5 kilograms” in the last year; and for maintenance of exercise, we got “whether respondent had spent at least 10 minutes for walking (*Walking_y_n*) and moderate activities (*Moderate_y_n*)” and “the minutes the respondent had spent during a usual week for walking (*Walking frequency*) and moderate activities (*Moderate frequency*).”

Covariates in our model mainly included demographic characteristics (age, gender, marital status, whether living in rural areas), education, wealth status, health insurance, health status and functioning, social contacts, and living information. For wealth status, we measured household total income. If a respondent had any kind of health insurance, we set the health insurance variable to be 1, otherwise 0. Current health status would be expected to influence the performance of health behaviors. We used mental health, physical functioning (PFs), activities of daily living (ADLs), instrumental activities of daily living (IADLs), “whether had body pain,” and “the number of disabilities” to measure health status of elderly people. We chose the diabetics and hypertension patients as our sample. In addition, we calculated the number of other chronic conditions as a control variable, including dyslipidemia, cancer or malignant tumor, chronic lung diseases, liver disease, heart attack, stroke, kidney disease, stomach or other digestive disease, arthritis or rheumatism, and asthma. For social contact control, we used the number of children and whether respondents participated in social activities. For living information, the distance between house and bus stop/train station and village/community economic status were included. We also controlled people’s drinking status in our model. The detail information about all the variables can be found in Additional file 1.

### Estimation methods

In order to control the selection bias between informal care, health behaviors, and confounding factors, we employed the propensity score matching method to get empirical results. We used both the nearest neighbor matching and full matching in our model. The treated group was elderly people with informal care, and the control group got elderly people without informal care. We could obtain the average treatment effects under certain assumptions [23] as the following:

$$ E\left({Y}_{i1}|{D}_i=1,\widehat{P}\left({X}_i\right)\right)-E\left({Y}_{i0}|{D}_0=0,\widehat{P}\left({X}_i\right)\right) $$

where *Y*_*i*_ is the health behaviors of the *i*-th individual, *D*_*i*_ = 1 and *D*_0_ = 0 denote that the individual was in the treated group and control group, respectively. *X*_*i*_ is the observable characteristics, and $\widehat{P}\left({X}_i\right)$ is the selected individual’s propensity score.

Missing data is a critical issue in survey data and deleting it blindly may lead to information loss. In this paper, we used a general multiple imputation method to fill all missing data.

## Results and discussion

### Descriptive analyses

Three thousand one hundred ninety-eight, 4466, 3352, and 3747 respondents were included in our research for smoking control, dietetic regulation, weight control, and maintenance of exercise, respectively. Table 1 shows the descriptive results. There were obvious differences between the cared group and non-cared group for each doctor’s advice. Elderly people with care were younger than those without care. The education and economic level were higher for elderly people with care. Cared people were more likely to buy health insurance. Health status of the two groups did not hold obvious differences.

**Table 1:** Means of characteristics of IC and non-IC individuals

	Smoking control		Dietetic regulation		Weight control		Maintenance of exercising	
	IC	Non-IC	IC	Non-IC	IC	Non-IC	IC	Non-IC
Age	60.87	62.45	61.60	62.40	60.43	62.49	61.25	62.49
Age 1 (60–75)	0.36	0.32	0.34	0.32	0.34	0.32	0.36	0.32
Age 2 (75–85)	0.15	0.19	0.17	0.18	0.14	0.19	0.16	0.19
Age 3 (> 85)	0.03	0.06	0.05	0.06	0.04	0.06	0.04	0.06
Gender	0.21	0.61	0.51	0.61	0.50	0.61	0.50	0.61
Marital status	0.88	0.83	0.86	0.82	0.88	0.83	0.86	0.82
Living in rural area	0.32	0.26	0.36	0.26	0.42	0.26	0.44	0.26
Education	0.65	0.38	0.58	0.38	0.72	0.38	0.69	0.38
Education 1 (middle school)	0.25	0.18	0.21	0.18	0.25	0.18	0.24	0.18
Education 2 (high school)	0.13	0.08	0.13	0.08	0.15	0.08	0.14	0.08
Education 3 (junior or college and above)	0.05	0.02	0.04	0.02	0.06	0.02	0.06	0.02
Total household income (RMB/thousands)	1456.30	847.18	925.34	873.3	1211.78	850.56	1006.99	857.09
Income 1 (2000, 7000)	0.29	0.28	0.28	0.28	0.28	0.29	0.28	0.28
Income 2 (7000, 20,000)	0.27	0.26	0.27	0.26	0.27	0.26	0.27	0.26
Income 3 (> 20,000)	0.17	0.15	0.18	0.15	0.19	0.15	0.19	0.15
Health insurance	0.96	0.93	0.95	0.93	0.94	0.93	0.95	0.93
Health status and function								
Mental health	16.60	16.76	17.16	16.66	16.78	16.76	17.01	16.76
PFs	2.31	2.57	2.59	2.55	2.44	2.57	2.58	2.56
ADLs	0.74	0.69	0.75	0.68	0.65	0.69	0.73	0.69
IADLs	0.62	0.70	0.72	0.68	0.62	0.69	0.71	0.69
Body pains	0.35	0.36	0.39	0.36	0.37	0.36	0.37	0.36
Disabilities	0.31	0.30	0.27	0.30	0.27	0.30	0.28	0.31
Other chronic diseases	2.70	2.33	2.76	2.28	2.82	2.32	2.80	2.31
Self-report health	0.15	0.16	0.13	0.16	0.13	0.16	0.13	0.16
Social contact								
Children	0.51	0.56	0.54	0.56	0.53	0.56	0.53	0.56
Social activities	0.83	0.74	0.85	0.73	0.88	0.74	0.94	0.73
Living information								
Distance to bus stop	2.60	2.96	2.69	2.96	2.05	2.92	2.28	2.93
Distance to train station	43.96	58.06	52.28	58.85	41.94	58.32	48.48	58.18
Village/community economic status	3.82	3.79	3.91	3.77	3.93	3.78	3.94	3.79
Drinking status	0.85	0.45	0.50	0.45	0.50	0.45	0.48	0.45
Observations	1068	2130	2400	2066	1231	2121	1642	2105

Notes: the baseline categories are “age < 60,” “marital status: unmarried,” “living in the rural area: village,” “gender: female,” “Education: primary school and below,” and “health insurance: no insurance”

*IC* informal care

Table 2 shows the means of characteristics for each doctor’s advice. Individuals with informal care were different with the control group in many ways. For smoking status, people with informal care had a worse smoking status except “the time lag between wake up and the first cigarette.” Being cared by others brought a better dietary habit which was measured by “the number of meals.” The informal cared group had lower BMI and the possibility of “losing weight”; however, there was no obvious difference in “gaining weight.” For exercise, people with informal care had a higher “possibility of walking,” but they had a lower “frequency of walking,” and the results were similar to moderate exercise.

**Table 2:** Means of characteristics of doctor advices

	Items	IC	Non-IC
Smoking control	Whether_smoking	0.32	0.11
	No.cigarette	18.00	18.22
	Wake smoking	1.31	1.37
Dietetic regulation	No.meals	2.68	2.64
Weight control	BMI	17.12	18.20
	Weight gain	0.03	0.03
	Weight loss	0.13	0.10
Maintenance of exercising	Walking_y_n	0.75	0.71
	Walking frequency	5.10	7.43
	Moderate_y_n	0.35	0.29
	Moderate frequency	2.32	3.84

Notes: the baseline categories are “whether smoking: no,” “gain weight: no,” “lose weight: no,” “walking_y_n: no,” and “moderate_y_n: no”

*IC* informal care

### Estimation results

Figures in Additional file 1 show the distribution of propensity scores across the treatment and control groups. The graphs showed that there was sufficient overlap between the cared and non-cared groups.

We used the propensity score matching method to reduce the possible influence from selection bias and confounding factors. Table 3 shows the results for both “Full Matching” (ATT^a^) and “Nearest Neighbor Matching” (ATT^b^). The results from the two methods were basically consistent. Regarding smoking control, people with care were more likely to smoke less (ATT^a^, *p* < 0.01; ATT^b^, *p* < 0.05) and had longer “time lag between wake up and the first cigarette” (ATT^a^, *p* < 0.05; ATT^b^, *p* < 0.05). With respect to the dietetic regulation, cared people ate more times per day (ATT^a^, *p* < 0.001; ATT^b^, *p* < 0.001). With regard to weight control, there were no significant differences between the two groups for BMI and “weight loss.” From our results, elderly people with informal care had a lower possibility for “gain weight” (ATT^a^, *p* < 0.05; ATT^b^, *p* < 0.05). Comparing the differences between the two groups for maintenance of exercise, we found that cared people were more likely to walk for exercise (ATT^a^, *p* < 0.05; ATT^b^, *p* < 0.05). However, there was no significant result for other exercise variables.

**Table 3:** The effects of informal care on health behaviors

	Matched sample		Mean		ATT^a^	Matched sample		Mean		ATT^b^
	IC	Non-IC	IC	Non-IC		IC	Non-IC	IC	Non-IC	
Smoking control										
No.smoking	294	232	18.295	21.425	−1.82** (1.721)	294	232	18.295	20.48	−1.53* (1.269)
Smoking in the morning	602	1244	0.350	0.493	−1.95* (0.073)	602	1244	0.350	0.418	−1.45* (0.046)
Dietetic regulation										
No.meals	1393	1187	2.933	2.853	3.67*** (0.021)	1393	1187	2.933	2.855	4.77*** (0.016)
Weight control										
BMI	705	1212	17.12	18.30	−1.55 (526.32)	705	1212	17.120	18.02	−1.35 (204.54)
Weight gain	136	263	0.029	0.088	−1.99* (0.029)	136	263	0.029	0.066	−1.74* (0.021)
Weight loss	135	263	0.118	0.089	0.70 (0.042)	135	263	0.118	0.098	0.51 (0.038)
Maintenance of exercise										
Walking_y_n	112	114	0.750	0.562	2.00* (0.093)	112	114	0.750	0.604	1.96* (0.074)
Walking frequency	80	75	11.9	9.50	0.96 (2.493)	80	75	11.9	9.535	1.10 (2.139)
Moderate_y_n	104	116	0.346	0.25	1.01 (0.094)	104	116	0.346	0.290	0.82 (0.073)
Moderate frequency	85	86	8.50	6.50	1.23 (2.230)	85	86	8.50	6.41	1.30 (2.112)

Notes: *significant at 10%; **significant at 5%; ***significant at 1%. Standard errors are in parentheses. All matching parameters are default. Italicized terms are used for emphasis

*IC* informal care, *ATT* average treatment effect for the treated

^a^The propensity score matching method is “Full Matching”

^b^The propensity score matching method is “Nearest Neighbor Matching”

## Discussion

Social support/control can regulate people’s health behaviors [6, 16], so we hypothesized that caregivers could supervise and persuade elderly people about their bad health behaviors by providing informal care. By focusing on the chronic diseases for which healthy behaviors are needed [9], we used the propensity score matching method to empirically estimate the effects of informal care on health behaviors of elderly people with chronic diseases in China. Our results showed that informal care generally could help improve health behaviors for elderly people from four aspects: smoking control, dietetic regulation, weight control, and maintenance of exercise.

For smoking control, our results indicated that being cared for by others helped elderly people decrease smoking intensity (measured by the amount of smoking daily) and lengthened “the time lag between waking and the first cigarette.” For dietetic regulation, diabetics need to have many meals, but less food at each. We did not have the data that can be used to measure how much respondents eat each time, so we can only compare “the number of meals per day.” Our results showed that cared people ate more times per day. For weight control, doctors often give advice on weight control for chronic disease patients. There was no significant result for BMI. It is hard for elderly people to reduce weight, and we also found that there was no significant influence of informal care on “weight loss.” “Weight gain” is a common health problem for elderly people, especially for people with chronic diseases. From our results, informal care decreased people’s probability of “5 kilograms weight gained.” Overeating is a high-risk behavior; it will bring big benefits for elderly people if informal care could prevent it. For exercise, we only got a significant result on “whether walk for exercise.” “Walking frequency,” “whether to have moderate exercise,” and “frequency of moderate exercise” did not show significant differences between the cared group and the control group. Although we hypothesized caregivers could supervise and persuade elderly people to improve their health behavior, body functions of elderly people degenerate gradually with age, and they could not afford intense exercise, which can be found in our empirical results. Informal care has been proven to be workable in improving health outcomes [5, 12, 25]; our results give empirical evidence to the role of family members in the regulation of health behaviors and appeal of raising the quantity of informal care.

## Conclusions

### Strengths and limitation

Our paper contributes to existing studies in several ways. First, our results are of significance to Grossman’s health capital theory and social support theories. Our results show that informal care is indeed an important source to produce health capital. Our study has once more proven that social support/control helps change individual behaviors. Second, informal care mainly comes from adult children and is an important source of care for elderly people with daily activities [7]. Existing studies have proved that elderly people with informal care have better health status which are measured by ADL, IADL, and diseases [31, 32], and most of the existing results come from developed countries. However, they do not provide insight on whether informal care can help improve health behaviors. To our knowledge, we are the first study to empirically research the role of informal care in improving health behaviors. Third, health behaviors are very important for patients with chronic diseases. There are quite a number of studies that have examined the effect of health behavior intervention on chronic disease management; however, they failed to research the health behavior improvement from informal care for patients with chronic diseases. Our study helps fill this gap. Fourth, our study enriches these studies that examine the impact factors of compliance of doctor’s advices and has proved that informal care can improve the compliance of doctor’s advice. Fifth, for the research data, we employ a new national household survey data that contains detailed information about health status, doctor’s advice, and informal care information which remedies many shortcomings of other data sets. Our results are more reliable. Last, we research our questions by studying elderly people in China who are facing the challenge of an aging population. Based on the above, a study investigating the Chinese population is needed.

There are several limitations of this paper. First, we do not distinguish the sources of informal care. The impacts of different sources of informal care on health behaviors may be different. For instance, we can guess that informal care from children is more effective for improvement of health behaviors than informal care from friends. Second, we do not measure the intensity of informal care which may influence the presentation of effects of informal care. However, our research has shown positive impacts of informal care on improvement of health behaviors, even only measuring informal care through a dummy variable. We suggest future research to explore the deep role of informal care.

### Implication

Our results show that informal care can improve health behaviors effectively. Since formal care is likely to be more expensive than informal care from the perspective of the health and social care budget, policy makers who aim to improve health status should take informal care of elderly people into account and provide appropriate policies to meet the demand of informal care. Our birth policy and postponed retirement policy may decrease the informal care supply, so adjustments of these policies are important and urgent to meet the current situation of the aged tendency of the population. In addition, we infer that formal care is also useful for improving health behaviors; the government could give more support to develop organizations to provide formal care, such as nursing homes. Moreover, we give suggestions to hospitals and villages/communities to encourage individuals to provide care to elderly people.

## Additional file

Additional file 1: Detailed results. (DOCX 65 kb)

## Abbreviations

ADLs: Activities of daily living
BMI: Body mass index
CHARLS: The China Health and Retirement Longitudinal Study
IADLs: Instrumental activities of daily living
PF: Physical functioning

## Footnotes

1 Data come from nutrition and chronic disease status reports among Chinese people which are issued by the National Health and Family Planning Commission of the People’s Republic of China in 2015 (http://mt.sohu.com/20150702/n416051446.shtml).

2 Data come from the National Bureau of Statistics of the People’s Republic of China in 2015 (http://www.stats.gov.cn/tjsj/pcsj/rkpc/6rp/indexch.htm).

3 Data come from nutrition and chronic disease status reports among Chinese people which are issued by the National Health and Family Planning Commission of the People’s Republic of China in 2015 (http://mt.sohu.com/20150702/n416051446.shtml).
