# Supplementary material for: Informal care and health behaviors among elderly people with chronic diseases
Source: J Health Popul Nutr. 2017 Dec 6;36:40. doi: 10.1186/s41043-017-0117-x (PMC5717826; doi:10.1186/s41043-017-0117-x)
Supplement: Additional file 1: — Detailed results. (DOCX 65 kb) [file 41043_2017_117_MOESM1_ESM.docx]

**Supplementary Materials**

**All variables are measured by the following questions:**

- Mental health (measured by the sum of scores):

Mental health was calculated as the sum of scores from the following 10 questions: bothered by things, had trouble keeping mind, depressed, everything respondent did was an effort, hopeful, fearful, poor quality of sleep, happy, lonely, and could not get “going”.

e.g. “I was bothered by things that don’t usually bother me”

(1) Rarely or none of the time (<1 day) (code 1)

(2) Some or a little of the time (1-2 days) (code 2)

(3) Occasionally or a moderate amount of the time (3-4 days) (code 3)

(4) Most or all of the time (5-7 days) (code 4)

- Physical Functioning, Activities of Daily Living and Instrumental Activities of Daily Living (measured by the sum of scores):

Whether respondent had difficult with Physical Functioning (PFs) (running about 1 km, getting up, climbing stairs, crouching, extending arms, carrying weights, and picking up a small coin), Activities of Daily Living (ADLs) (bathing, dressing, eating, using the toilet, getting into or out of bed, controlling urination, and walking 100 meters), and Instrumental Activities of Daily Living (IADLs) (shopping, cooking, managing money, taking medications, and doing housework) limitation.

e.g. “Do you have any difficulty with running or jogging about 1 km?”

(1) No, I don’t have any difficulty) (code 1)

(2) I have difficulty but can still do it (code 2)

(3) Yes, I have difficulty and need help (code 3)

(4) I cannot do it (code 4)

- Disability (measured by the sum of scores):

Disability was measured by “do you have one of the following disabilities: physical disabilities, brain damage/mental retardation, vision problem, hearing problem and speech impediment (choose all that apply)”.

- Other chronic diseases (measured by the sum of scores):

Other chronic diseases included dyslipidemia, cancer or malignant tumor, chronic lung diseases, liver disease, heart attack, stroke, kidney disease, stomach or other digestive disease, arthritis or rheumatism, and asthma.

- Self-reported health (measured by ordered variable)：

“Would you say your health is excellent, very good, good, fair, or poor?”

1. Excellent (code 1)
2. Very good (code 2)
3. Good (code 3)
4. Fair (code 4)
5. Poor (code 5)

**Histogram of propensity scores of treatment vs. control group for each doctor advic**e

Notes: the left figure is for “Full Marching” and right one is for “Nearest Neighbor Matching”.

- Smoking control

1. the number of cigarettes consumed in one day

1. the time lag between wake up and the first cigarette

- Dietetic regulation

1. the number of meals

- Weight control

1. BMI

1. Weight gain

1. Weight loss

- Maintenance of exercising

1. Walking exercise

1. Frequency of walking exercise

1. Moderate exercise

1. Frequency of moderate exercise
